# Impact of Rheology-Based Optimum Parameters on Enhancing the Mechanical Properties and Fatigue of Additively Manufactured Acrylonitrile–Butadiene–Styrene/Graphene Nanoplatelet Composites

**DOI:** 10.3390/polym16091273

**Published:** 2024-05-02

**Authors:** Soran Hassanifard, Kamran Behdinan

**Affiliations:** Advanced Research Laboratory for Multifunctional Lightweight Structures (ARL-MLS), Department of Mechanical and Industrial Engineering, University of Toronto, Toronto, ON M5S 3G8, Canada

**Keywords:** 3D printing, composite filaments, rheological properties, fatigue life

## Abstract

This study investigates the interaction between static and fatigue strength and the rheological properties of acrylonitrile–butadiene–styrene (ABS) polymer reinforced with graphene nanoplatelets (GNPs) in both filament and 3D-printed forms. Specifically focusing on the effects of 1.0 wt.% GNPs, the study examines their influence on static/fatigue responses. The rheological behaviour of pure ABS polymer and ABS/GNPs nanocomposite samples, fabricated through material extrusion, is evaluated. The results indicated that the addition of 1.0 wt.% GNPs to the ABS matrix improved the elastic modulus of the nanocomposite filaments by up to about 34%, while reducing their ductility by approximately 60%. Observations revealed that the static and fatigue responses of the composite filament materials and 3D-printed parts were not solely attributed to differences in mechanical properties, but were also influenced by extrusion-related process parameters. The shark-skin effect, directly related to the material’s rheological properties, had a major impact on static strength and fatigue life. The proposed method involved adjusting the temperature of the heating zones of the extruder during filament production to enhance the static response of the filament and using a higher nozzle temperature (270 °C) to improve the fatigue life of the 3D-printed samples. The findings reveal that the proposed parameter optimisation led to filaments with minimised shark-skin effects, resulting in an improvement in ultimate tensile strength compared to pure ABS. Moreover, the 3D-printed samples produced with a higher nozzle temperature exhibited increased fatigue lives compared to those manufactured under identical conditions as pure ABS.

## 1. Introduction

Several 3D printing techniques have been developed for fabricating plastic materials. The most commonly used 3D printing technique based on material extrusion is Fused Filament Fabrication (FFF), in which a plastic material in the form of a filament passes through a nozzle and, simultaneously, its temperature rises above its melting point. The exited extrudate is laid on the printer’s bed layer by layer with a desired raster angle and building direction to form the final workpiece. In the FFF method, several process parameters including layer thickness, nozzle diameter, printing velocity, nozzle and bed temperatures, raster orientation, and building direction can impact the quality of the part and, consequently, its mechanical properties [1,2].

The increasing popularity of additive manufacturing (AM) technologies has led to a focus on improving the physical and mechanical characteristics of parts made using these methods. Numerous investigations have been conducted to enhance the physical and mechanical properties of the parts produced using these techniques. Notably, the creation of nanocomposite materials by incorporating nano/micro fillers into the matrix during the printing process emerges as a prominent approach [3,4,5]. However, despite the potential benefits of using 3D-printed nanocomposite materials for sensitive applications, manufacturers face several processing-related challenges. These include the extrudate swelling effect, which is linked to the material’s rheological behaviour [6,7], and low adhesion between the printed layers [8], which can result in poor quality parts. Another phenomenon detrimentally affecting filament and FFF-processed 3D-printed quality is the shark-skin effect, characterised by surface irregularities and texture inconsistencies. This phenomenon can compromise the overall quality and mechanical integrity of the printed parts, leading to reduced functionality and structural integrity. Addressing the shark-skin effect is essential for ensuring high-quality 3D-printed components in various applications. Excessive nano-filler addition can increase the likelihood of the shark-skin effect occurring when the extrusion process parameters remain constant. The main issue arises from the significant rise in material viscosity as more nanofillers are added. This makes it harder for the material to flow smoothly through the nozzle during extrusion. Consequently, keeping the print quality consistent becomes more challenging, potentially resulting in defects in the final printed parts. To overcome these obstacles, a deeper comprehension of the relationship between the rheological and mechanical properties of 3D-printed nanocomposites is necessary. This understanding facilitates the enhancement of the reliability of these materials, making them suitable for more demanding applications.

The mechanical properties of 3D-printed plastic parts fabricated using the FFF technique are directly related to their rheological characteristics, which are fundamental in the 3D printing process. The rheological properties of a material can be characterised by its viscosity, which is influenced by factors such as temperature and shear rate [9,10]. It has been shown through several research studies that the melt rheology of the polymer used in material extrusion additive manufacturing impacts the printability and static and fatigue behaviour of the printed parts. He and Khan [11] studied the effects of nozzle diameter, layer height, and building direction on the flexural fatigue strength of 3D-printed ABS samples fabricated through the FFF technique. They also examined the variation in storage modulus (G′) with changing printing process parameters. They found that the storage modulus values impacted most with the change in building direction and nozzle diameter. They reported that samples fabricated with a larger nozzle diameter possessed higher values of mean storage modulus and, consequently, higher flexural fatigue strength. The addition of fillers to the matrix through 3D printing can also change the rheological properties of the nanocomposite. Larraza et al. [12] studied the filament property and printability of a polyurethane polymer reinforced with cellulose nanofibers and graphene. They also investigated the storage modulus and tanδ for the nanocomposite filaments. They reported that nanocomposite filaments containing graphene experienced higher values of storage modulus than those containing cellulose nanofibers. A similar trend was reported for 3D-printed nanocomposites as well. This increment in storage modulus could be attributed to the fact that graphene possesses an extremely high elastic modulus and large specific surface area. Vidakis et al. [5] studied the influence of the addition of silicon dioxide nanofiller into the polypropylene matrix through melt mixing extrusion operations on the mechanical performance and viscoelastic properties of the printed samples. They conducted dynamic mechanical analysis and melt flow index testing to evaluate the rheological properties of the 3D-printed nanocomposite samples. They reported that the addition of 1.0 wt.% and 2.0 wt.% silica nanofillers to the matrix stiffened the nanocomposite materials significantly; however, a 4.0 wt.% silica concentration had no major impact on the storage modulus of the nanocomposite sample. Wang et al. [13] explored how graphene enhances radiation resistance in epoxy resin composites. They made composites with 0.3 wt.% graphene oxide (GO) and reduced graphene oxide (Hh-RGO) via resin transfer moulding. By analysing the microstructure, free radical content, thermal stability, and mechanical properties before and after γ-ray irradiation, they found that both GO and Hh-RGO reduced free radical generation, enhancing radiation resistance. Graphene nanoparticles also enhanced thermal stability and mechanical properties by mitigating free radical damage to the crosslinked network. Smirnov et al. [14] added different concentrations of alumina ceramic powder to the PLA polymer through the wet processing technique and examined the rheological behaviour and printability of the fabricated filaments. They reported that the addition of 50–70 vol.% alumina powder increased the viscosity of the filament dramatically, resulting in an increased surface roughness of the filament and worse quality of the printed samples. Thumsorn et al. [15] investigated the effects of the addition of carbon fibre and copper powder to PLA matrix on the layer adhesion and mechanical characteristics of FDM-processed 3D-printed composite samples. They reported that increasing the printer’s bed temperature would increase the crystallinity of the PLA, leading to an improvement in storage modulus. However, additives caused faster solidification, which could lead to generating more voids in the samples, resulting in the lower layer adhesion of the 3D-printed parts.

Previous studies on the material characterisation of 3D-printed nanocomposites have primarily focused on either their mechanical or rheological behaviour [16,17]. However, few works have explored the interconnection between the rheological and mechanical properties of AM nanocomposite materials [18,19]. Specifically, research on the interrelation of the cyclic and rheological behaviours of 3D-printed nanocomposites is limited. The novelty of this study lies in its focus on identifying and optimising the most influential processing-related parameters, with the aim of enhancing the mechanical properties and fatigue resistance of 3D-printed nanocomposite parts. It has been shown that adding GNPs to the ABS matrix would result in higher viscosity values at all shear rates, potentially affecting material flow during the extrusion process. Consequently, adjusting the process parameters, such as temperature values and rotational speed, becomes essential for producing high-quality filaments. This study aims to validate the efficacy of the proposed parameter optimisation methods in addressing challenges posed by the shark-skin effect in filament production and 3D-printed samples. Through the optimisation of temperature settings in all zones of the extruder and utilising higher nozzle temperature, significant improvements in fatigue life were observed. These findings highlight the effectiveness of rheology-based optimisation in enhancing the overall quality and mechanical performance of 3D-printed components.

## 2. Experiments

### 2.1. Materials and Specimen

In this study, the materials utilised include ABS polymer (supplied by 3DXTECH) as the matrix and GNPs (obtained from ACS Material, LLC, Pasadena, CA, USA) as the nanofiller. Due to a dearth of information regarding the impact of incorporating GNPs into the ABS matrix via the material extrusion method on rheological properties and its correlation with fatigue performance, the aforementioned matrix and filler were selected for the current investigation. During the production of particulate composites, the occurrence of agglomeration poses a significant challenge, often resulting in a decrease in the mechanical properties of the composite. To mitigate the likelihood of agglomeration, researchers have proposed various methods, such as surface modification and dispersion techniques [20].

**Figure 1:**
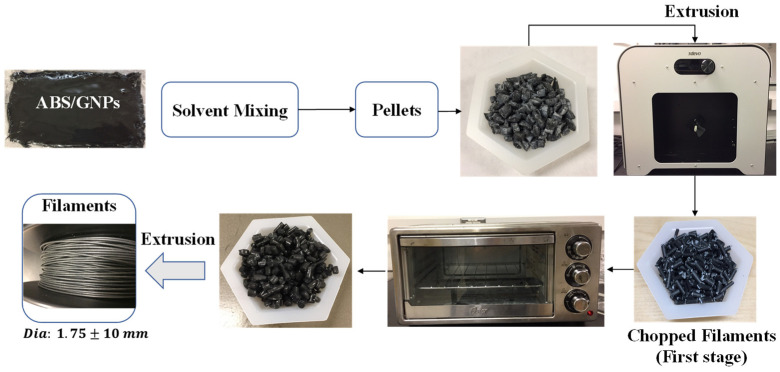
Nanocomposite filament fabrication process.

In this study, nanocomposite filaments with a concentration of 1.0 wt.% GNPs were fabricated using a combination of solvent and melt compounding methods. The fabrication process involved utilising a twin-screw 3Devo Composer 450 model filament maker with a nozzle diameter of 4 mm and a rotational speed ranging between 2 and 15 RPM. The chemical solvent method was employed to prepare the composite pellets. The mixing and extruding process to create filaments involved several steps to ensure the uniform dispersion of nanoparticles within the ABS matrix. Initially, pure ABS pellets were dissolved in acetone, followed by the addition of a well-mixed and sonicated solution of GNPs in acetone. This mixture was thoroughly homogenised to achieve a consistent nanoparticle distribution throughout the ABS matrix. Subsequently, the solution was dried to remove the solvent, and the resulting material was pelletised. To further enhance uniformity and minimise the possibility of nanoparticle agglomeration, an additional processing step was implemented. The pellets obtained from the initial mixing were used to produce the first batch of filaments. These filaments were then chopped, dried at 60 °C for four hours, and reintroduced as pellets for subsequent extrusion. This iterative process ensured greater uniformity in particle dispersion and minimised the occurrence of agglomerates, thereby improving the quality and properties of the final filament material. As a result, filaments with a diameter of 1.75 ± 0.1 mm were obtained. The entire filament fabrication process is illustrated in Figure 1.

**Figure 2:**
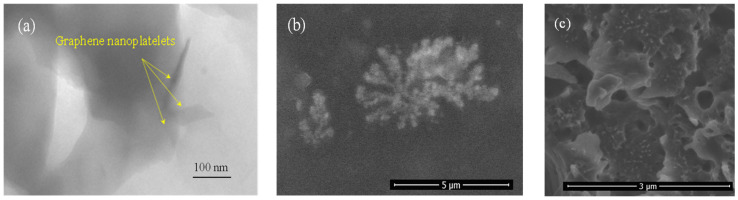
(**a**) TEM image of GNPs dispersed in ABS polymer, (**b**) an agglomeration of GNPs in the ABS matrix, and (**c**) image indicating well-dispersed GNPs in the matrix (small white dots).

**Figure 3:**
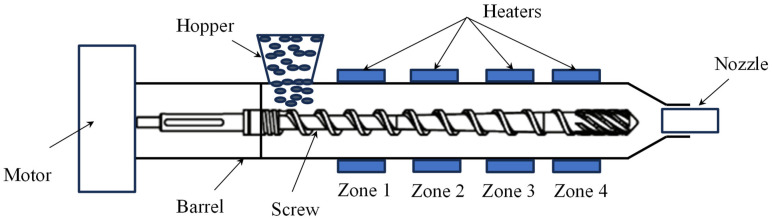
Schematic illustration of the heating section of the filament maker.

Figure 2 displays a Transmission Electron Microscopy (TEM) image revealing 2D morphology and well-dispersed graphene nano-platelets embedded within the ABS matrix, with no observable agglomeration. However, the application of the proposed method resulted in the occurrence of a phenomenon known as the “shark-skin effect”. This was primarily due to maintaining the same temperature settings across all heating zones of the extruder, consistent with those commonly used for fabricating pure ABS filament. Specifically, the extruder’s four heating zones were set to temperatures of 220 °C for the pressure build-up zone, 230 °C for the two middle zones, and 240 °C for the last zone (material exit from the nozzle), aligning well with recommendations from the literature [21]. Figure 3 provides a schematic illustration of the heating section of the filament maker, which consists of the above-mentioned four heating zones.

**Figure 4:**
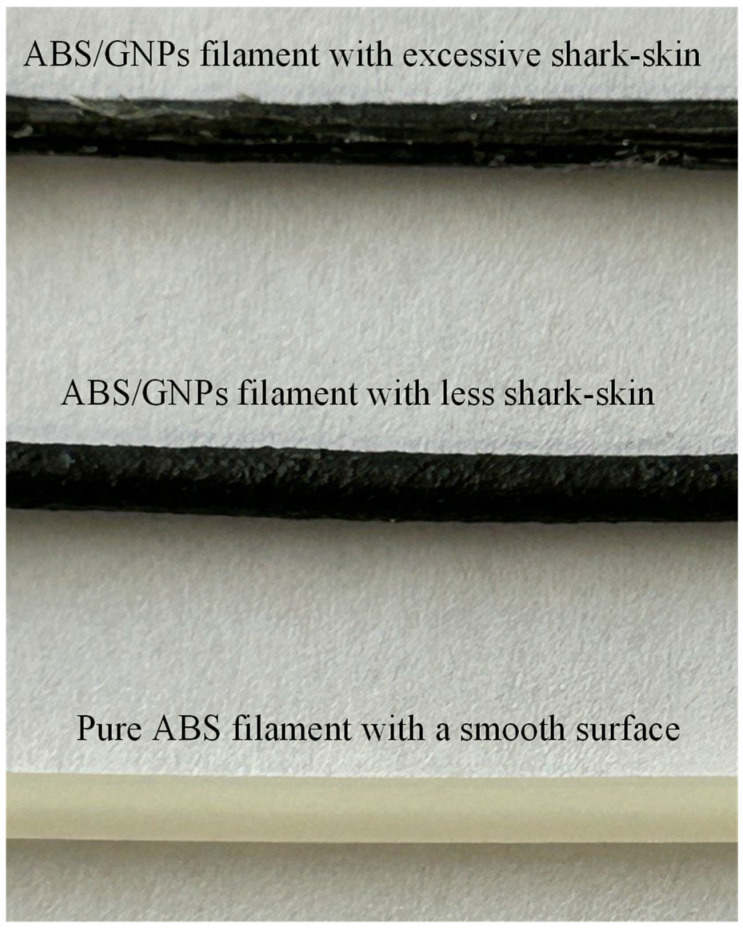
Surface images of the pure ABS and composite filaments.

Nevertheless, the addition of GNPs alters the viscosity of the material, thereby impeding its flowability and resulting in a filament with a rough surface texture. This surface irregularity has the potential to adversely affect the mechanical properties of the composite filament. Figure 4 illustrates the rough surface of the composite filament produced under the same temperature conditions as pure ABS, which yielded a smooth surface for the pure ABS filament. It is evident that the filament exhibiting excessive shark-skin formation has resulted in very poor surface quality, rendering it unsuitable for printing. Consequently, a marginally increased flow rate was employed to alleviate the shark-skin effect, yielding a filament of improved quality suitable for printing purposes. Despite the adjustment in flow rate, the persistence of the shark-skin effect is evident, suggesting the continued challenge of achieving optimal surface quality in composite filament fabrication.

In this study, the fabrication of nanocomposite 3D-printed samples was conducted using the widely recognised Prusa i3 MK3 3D printer, renowned for its versatility and performance in material extrusion printing. The printing process parameters employed for sample fabrication are detailed in Table 1. Flat 3D-printed samples were fabricated with three distinct raster orientations: 0°, 45°, and 90°, in accordance with the ASTM D7791-22 standard [22] for fatigue testing. The influence of raster orientation on the fatigue behaviour of the printed components was assessed by examining printing samples at these specific raster orientations.

**Figure 5:**
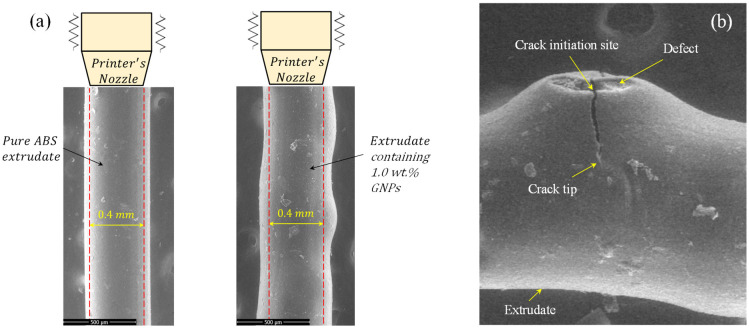
(**a**) Effects of die-swelling on the extrudate containing 1.0 wt.% GNPs and its comparison with pure ABS extrudate, (**b**) Defects and micro-cracks in the nanocomposite extrudate with 1.0 wt.% GNPs.

Die swelling, also called extrudate swelling, happens when a material expands after exiting the die or nozzle during extrusion. It is commonly observed in polymer processing and can affect the final product’s dimensions and surface quality, leading to defects or irregularities in the final product. This phenomenon is influenced by various factors such as material properties, processing conditions, and the presence of fillers. Figure 5 illustrates the impacts of incorporating 1.0 wt.% GNPs to the ABS matrix on (i) die-swelling and (ii) existing internal defects/micro-cracks when the extrudate is exited from the printer’s nozzle. As observed in Figure 5a, the die-swell effect is more noticeable in the case of nanocomposite extrudate containing 1.0 wt.% GNPs with an average value of die-swell equal to ~25% were compared to pure ABS extrudate with approximately 12% extrudate-swell. Figure 5b demonstrates that selecting inappropriate values for processing-related parameters may lead to the presence of internal defects in the extrudates. As seen in Figure 5b, a micro-crack has initiated from the vicinity of an internal defect in the extrudate with a 1.0 wt.% concentration of GNPs. For non-Newtonian materials such as viscoelastic polymers, normal stresses $\left(N_{1},\;N_{2}\right)$ appear in the direction perpendicular to the extrusion direction due to shear stresses $\left(\tau_{11},\;\tau_{22},\;\tau_{33}\right)$. These normal stress values escalate with increasing viscosity, resulting in an increase in acting force to potentially propagate the existing micro-cracks. The distorted extrudates, containing defects and micro-cracks, will result in low adhesion between the layers, warpage, rough surface, and poor quality of the printed samples.

The die-swell phenomenon was not observed in the filament-making process. This might be attributed to the fact that the rollers installed on the filament maker maintain the diameter of the filament at 1.75 mm using an optical sensor with an accuracy of 43 microns. In general, die-swell effects can be compensated for in several ways, such as optimising or re-designing the profile dies in polymer processing [23], or adjusting melt temperature and extrusion velocity to control the cooling rate in the material extrusion 3D printing process [6].

### 2.2. Thermal Analysis

**Figure 6:**
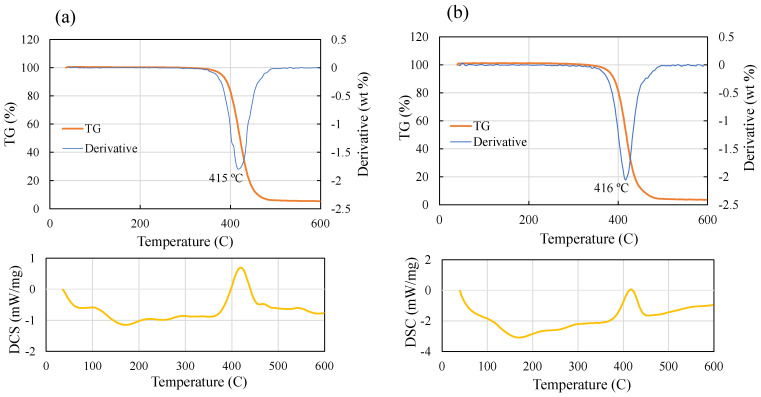
DSC and TGA analysis of (**a**) pure ABS, and (**b**) ABS/GNP composite material with a concentration of 1.0 wt.% GNPs.

Given that the extrusion of filaments using a twin-screw filament maker occurs within the temperature range of about 220–270 °C, and the nozzle temperature of the 3D printer ranges from 250 to 270 °C, conducting thermal analysis becomes imperative to ensure that material degradation has not happened within the operating temperature range. Additionally, it is crucial to examine how the incorporation of 1.0% GNP affects the thermal properties of the composite filament. Differential Scanning Calorimetry (DSC) and Thermogravimetric Analysis (TGA) were employed to conduct the thermal analysis. Figure 6 provides a comparison of the results. The analysis reveals no signs of material degradation within the specified temperature ranges. Moreover, the addition of 1.0% GNPs induces a slight forward shift in the glass transition temperature (T_g_) from approximately 105 °C (for pure ABS) to 110 °C. However, the degradation temperature remained unchanged at approximately 415 °C for both pure ABS and the composite material, which is far beyond the operation temperature for extrusion and the 3D printing process (220–270 °C). Throughout this range, no noticeable peaks were observed in the DSC graphs of the materials, suggesting that there were no significant changes in the crystallinity of ABS, and the addition of GNPs did not notably affect crystallinity.

### 2.3. Rheological Properties

**Figure 7:**
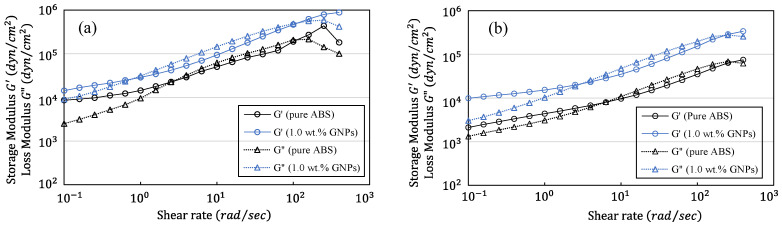
Storage modulus and loss modulus of ABS/GNP composite material with concentration of 1.0 wt.% GNPs and pure ABS at (**a**) 220 °C and (**b**) 250 °C.

Examining the rheological behaviour of ABS composites containing 1.0 wt.% GNP concentrations provides useful information about their mechanical performance and suitability for printing applications. To do so, rheology assessments were performed utilising a strain-controlled rheometer (ARES, TA Instruments, New Castle, DE, USA), which allowed for precise measurements of parameters to be taken including storage modulus, loss modulus, and viscosity. Frequency sweep oscillatory measurements were conducted via strain sweeps at 5% strain across a frequency range of 0.1–398 Hz. Rheology tests were conducted on both pure ABS and ABS/GNPs with a concentration of 1.0% GNP, evaluating G′ and G″ at two distinct temperatures, namely, 220 °C and 250 °C, which represent the typical range for extrusion processes. The analysis of the results, as depicted in Figure 7, reveals that both G′ and G″ values are notably higher for ABS/GNPs with a 1.0% GNP concentration compared to pure ABS, showing an increase of approximately 2.5 to 4.5 times across various shear rates. This observed trend signifies an enhancement in the viscoelastic properties and stiffness of the composite material with the incorporation of GNPs, indicating improved mechanical performance. As a result, 3D-printed components made from ABS/GNP composites could demonstrate improved strength and durability, rendering them ideal for demanding applications like structural components or load-bearing parts. It was also noted that increasing the temperature led to a reduction in the values of G′ and G″ for both pure ABS and ABS/GNPs; however, the trend remained consistent across different temperatures.

**Figure 8:**
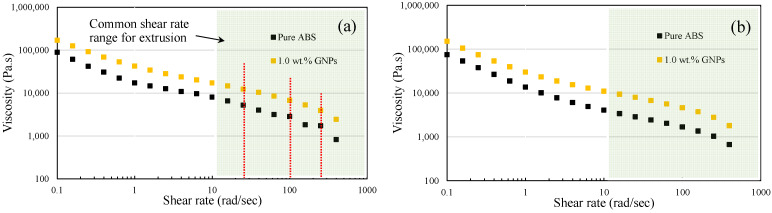
Viscosity versus shear rate for the ABS/GNP composite material with a concentration of 1.0 wt.% GNPs at (**a**) 220 °C and (**b**) 240 °C.

In the context of 3D printing, parameters such as printing temperature, extrusion rate, and filament viscosity are directly influenced by the rheological properties of composite materials. As demonstrated in Figure 8, shear thinning can be seen for both pure ABS and ABS/GNPs with 1.0 wt.% GNP composite material. It can also be observed that the viscosity of the ABS/GNP composite material with the concentration of 1.0 wt.% GNPs increases across all studied temperatures as compared to pure ABS, significantly impacting the flow characteristics and printability during the 3D printing process. This increase in viscosity necessitates adjustments such as higher temperature across the heating zones and higher rotational speeds for the extruder to ensure proper filament deposition and overall print quality.

**Figure 9:**
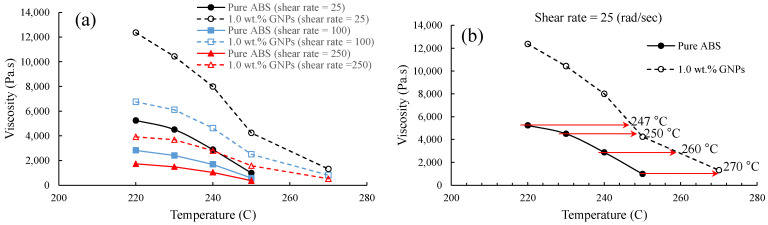
Viscosity versus temperature for pure ABS and the ABS/GNP composite material with 1.0 wt.% GNPs (**a**) at various shear rates and (**b**) details at shear rate of 25 rad/s.

Understanding the rheological properties of materials is important for optimising 3D printing parameters and attaining the desired mechanical characteristics in 3D-printed components. In the extrusion method, the typical shear rate ranges from 1.0 to 1000 rad/s, depending on the specifics of the process [24]. The twin-screw extruder utilised for filament fabrication has a rotation speed capability of up to 15 rpm. However, it has been recommended that for pure ABS, a rotational speed of 3.5 rpm yields optimal filament quality. As previously mentioned, to mitigate the likelihood of the shark-skin effect, while maintaining consistent temperatures across all four heating zones of the extruder, a rotational speed of 5.5 rpm was employed for fabricating the ABS/GNP composite filament. This adjustment aims to decrease viscosity and improve material flow, as evidenced by the filament with reduced shark-skin effect shown in Figure 4. Adjusting the rotational speed of the filament maker affects the shear rate experienced by the material during extrusion. Higher rotational speeds result in increased shear rates, which can reduce the viscosity of the material. Lower viscosity facilitates easier material flow through the extruder, potentially resulting in improved filament quality. Furthermore, enhancing the quality of the ABS/GNP composite filament was achieved through adjustments in the temperature values across the heating zones of the extruder. Figure 9 depicts the viscosity versus temperature for both pure ABS and the ABS/GNP composite material with a 1.0 wt.% GNP concentration. It is apparent that the viscosity values for the ABS/GNP composite material are approximately twice as high as those of pure ABS across all temperatures ranging from 220 to 250 °C and at all studied shear rates (Figure 9a). At a relatively low shear rate of 25 rad/s (Figure 9b), which roughly corresponds to a rotational speed of 5.5 rpm (based on a simplified equation $\dot{\gamma }=\pi DN/H$ in which $\dot{\gamma }$ is the shear rate, *D* is the barrel diameter, *N* is the rotational speed, and *H* is the channel depth [25]), the change in viscosity versus temperature indicates that a temperature shift of approximately 20 °C for the ABS/GNP composite material would result in viscosity values similar to those of pure ABS. The resulting filament exhibits a minimised shark-skin effect and is suitable for 3D printing. The characteristics of all discussed filaments are summarised in Table 2.

It is worth mentioning that increasing the GNP content in the matrix would result in higher viscosity values at all shear rates. This can affect the material flow during the extrusion process. Depending on the viscosity values, adjusting process parameters including temperature values and rotational speed can lead to high-quality filaments.

### 2.4. Tensile and Fatigue Tests

Three types of filaments mentioned in Table 2 (all filaments excluding Filament 2) were tensile tested and their ultimate tensile strength, Young’s modulus, and elongation were recorded and compared to each other. Quasi-static tests on the filaments and 3D-printed samples were carried out using a 5900-series Instron testing machine at a 1 mm/min rate, and the stress–strain curves were extracted. At least five samples were tested from each series of specimens.

Both filament types mentioned in Table 2, Filament 3 and Filament 4, were employed in fabricating 3D-printed samples. The samples made from Filament 3 were printed at a nozzle temperature of 250 °C, while those made from Filament 4 were printed at 270 °C. Each batch of samples was printed at three different raster orientations: 0°, 45°, and 90°. Subsequently, the printed samples underwent static and fatigue testing, and the results were then compared. Flat dog-bone shaped samples, characterised by a narrow section width of 10 mm, a length of the narrow section of 50 mm, and a sample thickness of 2.8 mm, were employed for both static and fatigue tests.

As illustrated in Figure 9b, the viscosity of the ABS/GNPs nanocomposite material with a 1.0 wt.% GNP concentration at a temperature of 270 °C and a shear rate of 25 rad/s closely mirrors that of ABS at 250 °C, the designated temperature for 3D printing pure ABS samples. Increasing the nozzle temperature from 250 °C to 270 °C would reduce the die-swell effect, resulting in better quality of the extrudate and 3D-printed samples. Fatigue tests of the samples were conducted at a frequency of 0.5 Hz and a stress ratio of zero to prevent buckling. A minimum of three samples were tested at each load level, and the maximum stresses were plotted against fatigue lives. The fatigue test results of the composite samples fabricated with the nozzle temperature of 270 °C were then compared with those fabricated under the same conditions as pure ABS.

## 3. Results and Discussion

**Figure 10:**
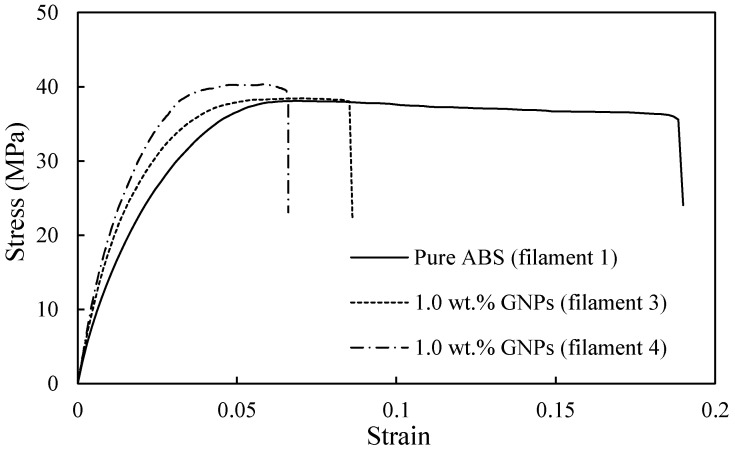
Stress–strain curves of the pure ABS and both ABS/GNP filaments with the concentration of 1.0 wt.% GNPs (Filaments 3 and 4 mentioned in Table 2).

Figure 10 presents the stress–strain curves of the ABS/GNP composite filaments containing 1.0 wt.% GNPs, which were fabricated with the adjustments aimed at reducing the shark-skin effect (Filaments 3 and 4 in Table 2), alongside that of pure ABS. The variations in ultimate tensile strength (UTS) and Young’s modulus were evaluated. As depicted in Figure 10, Filament 4 exhibited the highest UTS, as expected due to the minimised shark-skin effect, demonstrating an 8% improvement compared to pure ABS. The addition of 1.0 wt.% GNPs to the ABS matrix is anticipated to enhance UTS and Young’s modulus, given the excellent strength and stiffness properties of GNPs. However, Filament 3 did not show significant improvement in UTS compared to pure ABS, attributable to its poor filament quality and rough surface. Although the addition of 1.0 wt.% GNPs (Filament 4) led to a 34% increase in Young’s modulus, the ductility of the composite material decreased notably. Table 3 summarises the mechanical properties of the studied filaments.

The addition of GNPs to ABS was aimed at improving its mechanical properties, such as static/fatigue strengths and stiffness. If a filament with a higher percentage of GNPs could be fabricated using optimal parameters and if a shark-skin-free filament could be achieved, it would most likely possess a higher UTS. However, under the same conditions as pure ABS, the higher UTS for the filament with a concentration of 2.0 wt.% GNPs was not observed; instead, very brittle behaviour was reported [26]. This underscores the importance of optimising parameters to minimise the shark-skin effect while fabricating filaments.

Table 4 summarises the tensile test results of 3D-printed samples fabricated with Filaments 1 (pure ABS), 3, and 4 (nanocomposite filaments). The values of UTS, elastic modulus, and strain at breaks (%), were included. The results reveal that samples fabricated with Filaments 3 and 4 possess slightly higher UTS at all raster orientations. Also, adding GNPs to the ABS matrix resulted in higher elastic modulus at all raster angles. However, samples manufactured with Filament 4 possessed the least elongation. This was expected, as the latest filament experienced the least ductility as compared to other filaments. Upon reviewing the UTS data in Table 4, it becomes apparent that all 3D-printed samples exhibited anisotropic behaviour.

**Figure 11:**
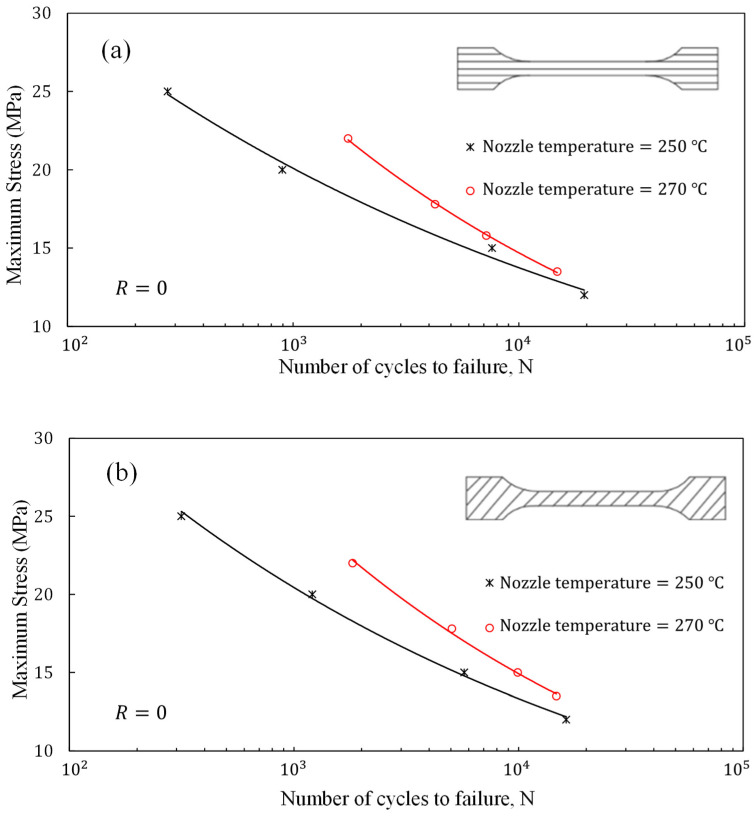

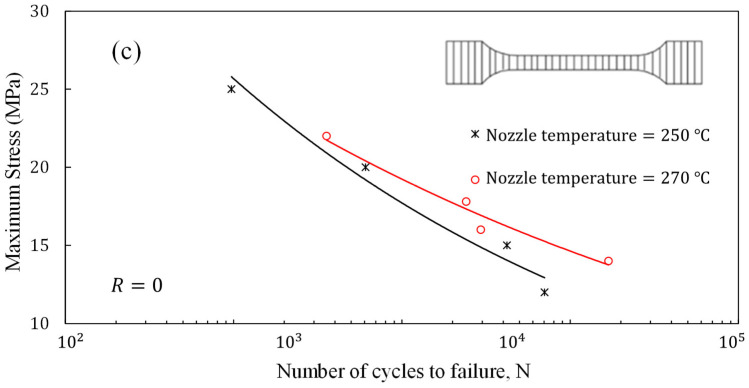
Comparison of fatigue life of ABS/GNP 3D-printed nanocomposite samples fabricated using nozzle temperatures of 250 °C and 270 °C at (**a**) 0°, (**b**) 45°, and (**c**) 90° raster orientations.

Figure 11a–c illustrate the S-N curves of the 3D-printed ABS/GNP composite specimens with 1.0 wt.% The GNP content was fabricated using nozzle temperatures of 250 °C and 270 °C at different raster orientations. It is evident that increasing the nozzle temperature results in improved fatigue life across all raster orientations. In particular, for the 0° and 45° raster orientations, this improvement is more pronounced in the low-cycle fatigue regime, whereas for samples with a 90° raster orientation, the modification in nozzle temperature leads to significant enhancement in fatigue life in the high-cycle fatigue regime. Overall, the enhancement in fatigue life can be attributed to the reduction in viscosity and the ease of material flow, resulting in higher sample quality. This underscores the importance of optimising parameters such as temperature range within the extruder and nozzle, extruder’s rotational speed, and nozzle temperature to achieve ideal sample quality. An excess of viscosity in the material can result in the unfavourable occurrence of the shark-skin effect during the printing process. Conversely, if the viscosity is too low, it becomes challenging to control the flow rate effectively. Therefore, achieving an optimal balance between viscosity levels is necessary to ensure high-quality 3D-printed samples with smooth surfaces.

## 4. Conclusions

The study examined the interaction between static and fatigue strength and the rheological properties of ABS polymer reinforced with GNPs in filament and 3D-printed forms. Through comprehensive experimentation and analysis, several key findings have emerged:

- The shark-skin effect, closely linked to the material’s rheological behaviour, emerged as a significant factor adversely impacting static strength and fatigue life. Optimising the temperature of all four heating zones of the extruder for filament production and utilising higher nozzle temperature (270 °C) for 3D-printed samples were proposed to address this challenge.
- The addition of 1.0 wt.% GNPs to the ABS matrix and a modification made to the temperature of the extruder’s heating zones significantly enhanced the elastic modulus and UTS of the nanocomposite filaments. In particular, the proposed parameter optimisation led to filaments with minimised shark-skin effects and an 8% higher UTS and 34% higher elastic modulus compared to pure ABS.
- The 3D-printed samples produced with a higher nozzle temperature exhibited increased fatigue lives at all raster orientations compared to those manufactured under identical conditions as pure ABS.
- The research underscores the critical importance of optimising parameters such as temperature range, the extruder’s rotational speed, and printer nozzle temperature to achieve ideal sample quality. Achieving a delicate balance between viscosity levels is vital in ensuring high-quality 3D-printed samples with smooth surfaces.

## Figures and Tables

**Table 1:** Printing process parameters used to fabricate the samples.

Layer height (mm)	0.2
Printing speed (mm/sec)	20
Infill density (%)	100
Nozzle diameter (mm)	0.4
Nozzle temperature (°C)	250, 270
Bed temperature (°C)	110

**Table 2:** The extrusion detail of pure ABS and ABS/GNP composite filaments.

Filament Type	Temperatures (°C)				RPM	Surface Condition
	Zone 1	Zone 2	Zone 3	Zone 4		
Filament 1 (pure ABS)	220	230	230	240	3.5	Excellent
Filament 2 (containing 1.0 wt.% GNPs)	220	230	230	240	3.5	Excessive shark skin
Filament 3 (containing 1.0 wt.% GNPs)	220	230	230	240	5.5	Less shark skin
Filament 4 (containing 1.0 wt.% GNPs)	247	250	250	260	5.5	Good

**Table 3:** Mechanical properties of the studied pure ABS and composite filaments.

Filament Type	UTS (MPa), SD	Young’s Modulus (MPa)
Filament 1 (pure ABS)	36.9, 2.1	1642
Filament 3 (containing 1.0 wt.% GNPs)	37.8, 1.2	2095
Filament 4 (containing 1.0 wt.% GNPs)	39.9, 2.4	2203

**Table 4:** Tensile test results of 3D-printed samples fabricated with Filaments 1, 3, and 4.

3D-Printed Samples	0° Raster Angle			45° Raster Angle			90° Raster Angle		
	UTS (MPa)	E (MPa)	Strain (%)	UTS (MPa)	E (MPa)	Strain (%)	UTS (MPa)	E (MPa)	Strain (%)
Fabricated with Filament 1	32.4	1522	5.6	32.9	1591	5.9	29	1489	3.9
Fabricated with Filament 3	34.4	1556	5.5	34.2	1702	4.8	29.8	1549	4.1
Fabricated with Filament 4	35.5	1772	3.8	34	1733	4.4	30	1639	2.7

## Data Availability

Data are contained within the article.
